# Impacts of Low Atmospheric Pressure on Properties of Cement Concrete in Plateau Areas: A Literature Review

**DOI:** 10.3390/ma12091384

**Published:** 2019-04-28

**Authors:** Jinyang Huo, Zhenjun Wang, Huaxin Chen, Rui He

**Affiliations:** 1 School of Materials Science and Engineering, Chang’an University, Xi’an 710061, China; 2017231039@chd.edu.cn (J.H.); heruia@163.com (R.H.); 2 Engineering Research Center of Pavement Materials, Ministry of Education of P.R. China, Chang’an University, Xi’an 710064, China

**Keywords:** plateau areas, low atmospheric pressure (LAP), properties of cement concrete, air entraining agent (AEA), bubble characteristics, improvement techniques

## Abstract

Low atmospheric pressure (LAP) can enormously affect properties of cement concrete in plateau areas. There are fewer studies and attendances on this issue than those of cement concrete in normal atmospheric pressure (AP), because of the limitations of both environmental conditions and instruments. In order to improve properties of cement concrete under LAP, influences of LAP on properties of cement concrete were reviewed in this work. The influence rules and mechanism on properties of cement concrete were summarized. The corresponding mechanism and techniques were put forward for enhancing the properties of cement concrete. The results of researchers show that LAP can significantly reduce the air entraining ability of the air entraining agent (AEA). Air content in concrete linearly decreases with the decrease of AP when other conditions are constant. If the initial air content is high, the decrease rate of air content increases with the decrease of AP. When the initial air content in cement concretes is similar, the greater the slump of cement concrete, the stronger its resistance to the decrease of air content caused by the decrease of AP. In addition, the condition of the bubble characteristics of hardened cement concrete under LAP is worse than that under normal AP. Therefore, the change of concrete properties under LAP is mainly attributed to these bubble characteristics, such as air content, bubble spacing coefficient, bubble radius and bubble specific surface area. In this work, nano-silica (negative charges) with cationic oligomeric surfactants is recommended as a new type of AEA to optimize the bubble characteristics under LAP in plateau areas.

## 1. Introduction

The altitude of plateau areas is high, and the air in these areas is thin, which forms a low atmospheric pressure (LAP) environment in these plateau areas. Within an altitude of 3000 m, the atmospheric pressure (AP) decreases by 1 mm Hg (about 133 Pa) for each 12 m increase. It is not a linear relationship in which the altitude of the plateau area is higher than 3000 m. But the AP decreases with the increase of the altitude. Therefore, the AP decreases unevenly with the altitude; and the higher the altitude is, the slower the AP decreases. The relationship between AP and altitude is shown in Equation (1).

(1) $$P=p_{0}\times {\left(1-H/44330\right)}^{5.225}$$

where, *P_0_* (kPa) is the normal AP (101.325kPa) and the *P* (kPa) is the AP value for altitude of *H* (m).

Bubbles have great influences on microstructures and macroscopic properties of cement concrete [1,2]. The property changes of concrete under LAP are closely related to the bubble characteristics [3,4,5,6,7,8,9,10,11]. Generally speaking, bubbles are mainly introduced by stirring, vibrating or adding an air entraining agent (AEA). Stirring and vibrating are simple physical ways. Adding AEA is a complex physical-chemical method, which includes the chemical method of using AEA to reduce the surface tension of the liquid along with the physical method of entraining and intercepting a large number of bubbles in the cement concrete mixing process [12,13,14,15,16,17,18,19,20,21,22]. In addition, the molecules of AEA react or adsorb with the molecules (or ions) produced by cement hydration and cement particles. Fagerlund [22] and Ley, et al. [23,24] studied the formation mechanism of bubbles in fresh cement concrete, and the phenomena of their amalgamation and cracking before the setting and the hardening of cement concrete, respectively. At the same time, some researchers also discussed the influence of different molding methods [25], superplasticizer [26,27], mineral admixtures [28,29], concrete mixing methods [30] and molding vibration time [31] on the formation and stability of bubbles in cement concrete. Therefore, there are many factors affecting the bubble characteristics. It can be seen that the LAP affects the properties of cement concrete through bubbles.

In this work, the influence rules and the mechanism of LAP on AEA and bubbles in cement concrete were summarized at first, and then the microcosmic and macroscopic changes of workability, mechanical property and durability of cement concrete were deeply analyzed according to the changes of the bubble characteristics. In view of these influences, corresponding improvement techniques and some opinions on the mechanism for developing new AEA were put forward, which were beneficial to the development of any new AEA. This work can put forward theoretical and technical supports for improving the properties of cement concrete under LAP for its wide applications in construction and building materials in plateau areas.

## 2. Influence of LAP on Bubble

### 2.1. Influence of LAP on Effects of AEA

Bubbles introduced by physical means are mostly unstable, which is more evident under LAP. Therefore, it is necessary to add AEA in concrete. The foaming capacity of AEA depends mainly upon its effect of reducing the surface tension of the solution, its diffusion properties and the elastic characteristics. The foam stability of AEA is equally important. The Gibbs-Marangoni theory can explain the role of AEA in foam film formation and persistence [21].

Zhu, et al. [3] determined the influences of AP on effects of AEA with the foaming test (Table 1 [3]) and the concrete mixture property test. In the foaming test, the normal AP in the Beijing area is regarded as the standard AP; and the AP in the Golmud area is only equivalent to 70% of normal AP. As shown in Table 1 [3], AP has influence on the particle size of the bubble, as well as the bubble stability. From the concrete mixture property test, it is found that the air content of the two places in Beijing and Golmud varies greatly. According to the above two experiments, the two experimental conclusions have a good correlation. The air content in concrete is low in the Golmud area. It proves that air content in concrete is closely related to AP. The AP has a great influence on air content in concrete. Under the same conditions (the type of AEA is SJ-2; the dosage of AEA is 0.2 g), the air content of concrete under LAP is lower than that under normal AP.

**Figure 1:**
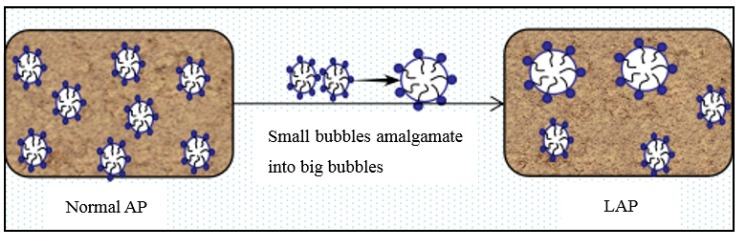
Schematic diagram of bubble amalgamation.

**Figure 2:**
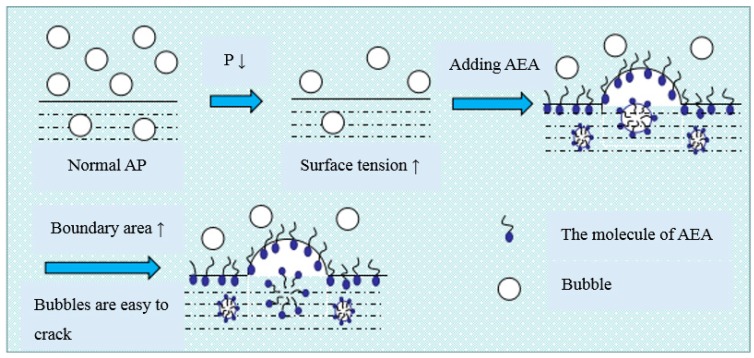
Schematic diagram of bubble crack.

LAP can weaken interfacial activity and foam stability of AEA [32]. In the cement-water-air system, the adsorption of AEA molecules on the interface of each phase decrease [21]. For AEA, it is not easy to introduce a large number of tiny bubbles. The thickness and strength of the hydration film on the surface of the bubble introduced by an AEA decrease, which makes the bubble easy to escape, amalgamate (Figure 1) and crack (Figure 2). In addition, LAP increases the surface tension of the liquid-air; and the foaming is a process that makes the interface area increase [13,15]. This inevitably leads to a great increase in the free energy and the instability of the thermodynamics, which makes bubbles shrink and crack, and decreases the air content in cement concrete.

**Figure 3:**
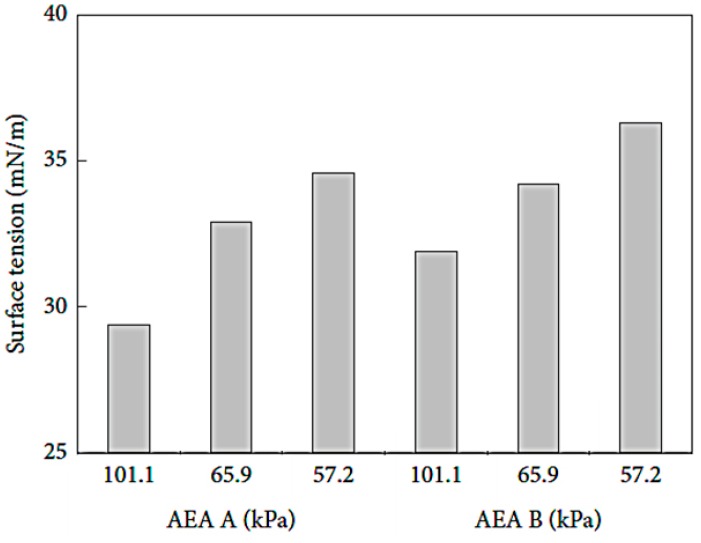
Surface tension histogram of air entraining agent (AEA) under different atmospheric pressure (AP).

**Figure 4:**
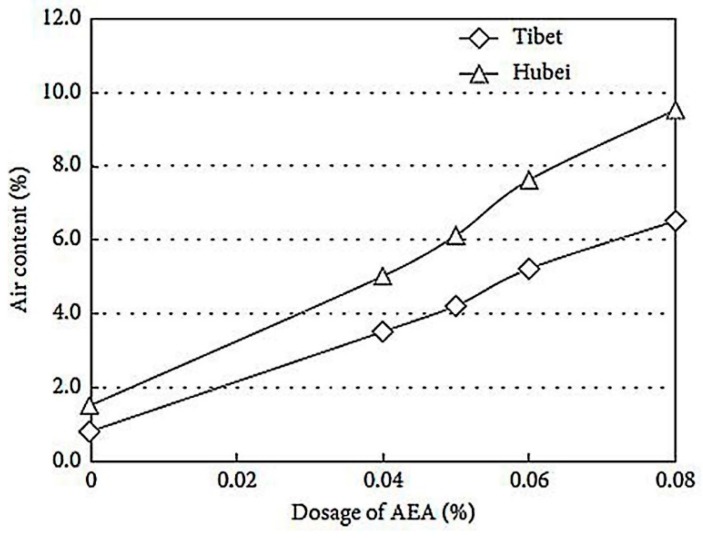
The relationship between dosage of AEA and air content under different AP.

Yan, et al. [32] studied the effect of AP on AEA. Their results are similar to those of Zhu, et al. [3]. As shown in Table 2 [32] and Figure 3 [32], the surface tension and the bubble morphology of different AEA under different AP are compared. When the AP reduces from 101.1 kPa to 57.2 kPa, the surface tension of the synthetic anionic surfactant and the alkyl sulfonate air entraining agent liquid both increase. The growth rate is 118% and 114%, respectively. Their foaming ability is reduced; the introduced bubbles have different sizes; the bubble film is thin; and the defoaming time is short. As shown in Figure 4 [32], the air content in concrete in the Tibet area (less than 70.0 kPa) is lower than that in the Hubei area (about 101.1kPa), when the same AEA dosage is introduced. Molecules at the liquid-air interface under LAP are obviously subjected to higher forces than that under normal AP, and the ability of AEA to reduce surface tension is reduced, which can decrease the air content in cement concrete.

Li, et al. [4,5,33] studied the effects of different AP on the AEA by simulating different AP in the plateau area. LAP can significantly reduce the air entraining ability of AEA. If other conditions are constant, the air content of concrete decreases linearly with the decrease of AP.

For example, in Table 3 [33], when AP is reduced to 50 kPa, the air content of concrete is reduced by about 20% to 49%. As shown in Table 4 [33], when the initial air content in concrete is higher, the air content decrease rate is higher with the decrease of AP. Its resistance to air content reduction also increases when the slump of concrete increases. Through this conclusion, we know that the increase of concrete slump can make AEA mix with the mixture evenly at the similar initial air content, and it is beneficial for AEA to make bubbles stable. Therefore, the ability of cement concrete to resist the decrease of air content becomes stronger. Among them, the initial air content is mainly produced by physical stirring. The stability of bubbles produced by physical stirring is poor, and the stability of bubbles is even worse, due to LAP. Li, et al. [8] conducted a test on the porosity change of concrete under LAP. They proved that LAP reduced the air entraining ability of AEA, which led to the decrease of air content in concrete. The results of Ke, et al. [9,10] are consistent with those of Li, et al. [8]. According to the studies of the above researchers, this work is summarized in Table 5. To sum up, the LAP significantly weakens the air entraining ability of AEA, which affects the air content of cement concrete.

### 2.2. Bubble Characteristics under LAP

#### 2.2.1. Changes of the Bubbles under LAP

**Figure 5:**
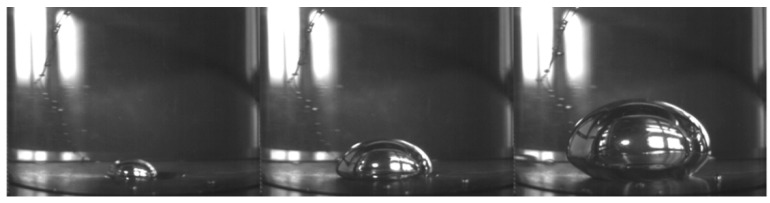
The growth and departure behavior of an isolated bubble under LAP.

Some studies showed that bubbles were no longer spherical [34,35,36], but flat spherical bubbles under LAP, as shown in Figure 5 [34]. They found that both the bubble growth time and departure radius increased with the decrease of AP through experiments of water, which means the lower vapor density and stronger surface tension under LAP. In addition, with the increase of the superheat on the bubble wall, the thermal boundary layer becomes thicker, and leads to the increase of the bubble departure diameter. The condensation effect and the Marangoni flow hinder the bubble growth, leading to the smaller bubble size under cooling conditions [37,38,39,40,41,42,43]. In the process of bubbles migrating outward, the condensation effect and the Marangoni flow hinder the growth of bubbles, resulting in a rapid decrease in the radius of the bubbles. In this unbalanced state, bubbles are easy to crack, which affects the bubble characteristics.

#### 2.2.2. Influences of LAP on Bubble Characteristics

The bubble characteristics mainly include air content, the bubble spacing coefficient, bubble radius and bubble specific surface area. Equations are as follows:

(2) a=S/N

where, the *a* is average bubble area; the *S* (μm^2^) is accumulated bubble area; the N is the number of bubbles.

(3) $$A_{s}=100na$$

where, the *A_s_* is air content, and the *n* is the number of bubbles per unit area.

(4) $$d=\frac{1}{N}\overset{N}{\underset{i=1}{\sum }}\left(2\sqrt{S_{i}/\pi }\right)$$

where, the *d* is average bubble diameter, and the *S_i_* (μm^2^) is bubble area.

(5) $$\alpha =\sqrt{6\pi /a}$$

where, the *α* is specific surface area.

(6) $$L=\{\begin{array}{l} P/\alpha A_{s},P/A_{s}<4.33\\ \left(3/\alpha \right)\left[1.4{\left(P/A_{s}+1\right)}^{1/3}-1\right],P/A_{s}\geq 4.33 \end{array}$$

where, *L* is the bubble spacing coefficient, and *P* (%) is paste content (volume fraction).

Li, et al. [6] studied the effect of LAP on pore structure with an LAP test chamber. The results are shown in Table 6 [6] and Table 7 [6]. The condition of pore structure characteristics of hardened concrete under LAP is lower than that under normal AP (the number of bubbles per unit volume decreases; the average diameter and specific surface area of bubbles increase; and the spacing coefficient of bubbles increases). 

When the air content of concrete reaches a constant, the spacing coefficient of a concrete bubble can reach 200 μm–300 μm under LAP. Li, et al. [7] studied the effect of AP reduction under different mix ratio and initial air content on the air content and bubble stability of concrete. The results show that the bubble stability of concrete decreases under LAP. The time loss of air content increases, and extending the vibration time leads to more bubble loss. These factors increase the spacing coefficient of cement concrete under LAP.

The properties of the liquid interface have changed under LAP. The bubble film becomes thin, and the strength is insufficient, which makes the bubbles remaining in concrete decrease significantly. At the same time, the whole system is in the unstable state of thermodynamics. In order to achieve a stable state, the interfacial area of each phase is automatically contracted. The bubble amalgamates to reduce its specific surface area, which increases the average radius and spacing coefficient of the bubble. Ma [11] conducted a LAP and low humidity maintenance test. The AP is changed when the humidity (H) is a constant value. The results are shown in Table 8 [11]. The porosity and average pore size of concrete increase with the decrease of AP.

The moisture evaporation in fresh concrete can increase during the setting and hardening process of concrete under LAP. The outward migration of moisture is accelerated due to the evaporation of moisture and the humidity field formed by the difference in humidity between the inside and the outside. This causes bubbles to migrate and crack with moisture movement. As a result, the air content decreases; the average radius and spacing coefficient of the bubble increases; the capillary pores increase; the porosity increases, and the average pore size increases.

**Figure 6:**
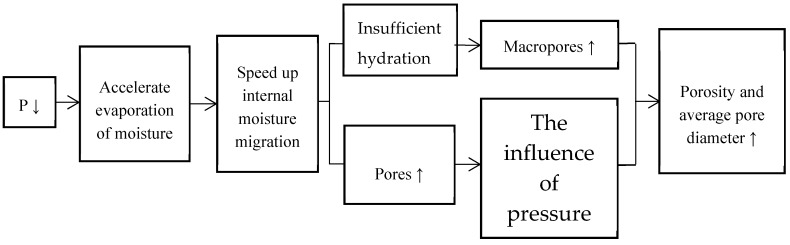
Schematic diagram of pore change.

In addition, the evaporation of moisture on the surface of hardened concrete is accelerated during curing. Therefore, it accelerates the internal moisture migration, which results in incomplete hydration, and the increases of macropore porosity and average pore size (Figure 6). To sum up, the changes of the bubble characteristics under LAP are: The number of bubbles per unit volume becomes small; the average diameter and the specific surface area of the bubble increase; and the spacing coefficient of the bubble increases. Thus, the bubble becomes unstable in a cement concrete mixture.

## 3. Influence of LAP on Properties of Cement Concrete

### 3.1. Influence of LAP on Workability of Cement Concrete

#### 3.1.1. Slump of Concrete Mixture

Ke, et al. [9] showed that the effect of AEA on concrete slump was small under LAP. The slump of concrete with different components under LAP was greater than the slump of concrete with different components under normal AP. The surface tension value of the liquid under LAP increases, which suppresses the foaming ability of AEA. This limits the effect of AEA on the concrete slump. Even if the dosage of AEA increases, there is no significant change in slump. If effects of AEA are not considered, the increase of the slump can be due to the viscosity of the concrete mixture under LAP. Han, et al. [44] showed that the slump of concrete increased with the increase of air content. According to the data, the air content of concrete under LAP has significantly changed. Therefore, the work considers that the slump of concrete under LAP is greatly influenced by its air content. Bubbles in concrete are like tiny glass beads in fly ash, which can influence the sliding friction coefficient and lubrication action between cementitious materials and aggregates.

#### 3.1.2. Slump Flow of Concrete Mixture

Ke, et al. [9] showed that under LAP, the effect of AEA on slump flow of concrete was small. The slump flow of concrete with different components under LAP was greater than that of concrete with different components under a normal AP. The main reason is similar to the concrete slump studied by Ke, et al. [9]. LAP greatly restricts the air entraining effect of AEA, which causes the effect of AEA on concrete slump flow to become small. The viscosity of the mixture is affected by the deterioration of the concrete under LAP. Concrete mixture is easy to segregate, which can increase the slump flow of concrete mixture.

#### 3.1.3. Pumpability of Concrete Mixture

Generally, the AP reduces about 10% for every 1000 m increase in altitude, as shown in Equation (1). The property of pumping concrete can inevitably change at high altitude areas [3]. In most cases, when the air content in concrete under LAP reduces, the pumpability of any concrete mixture also decreases.

Ke, et al. [9] showed that the air content of concrete decreased under LAP. The average pump pressure of concrete with different components increased largely, which made the pumpability decrease. Some bubbles can reduce the plastic viscosity to achieve an optimum value. The slump value of concrete decreases slightly. However, under LAP, the slump is affected, and the air content decreases greatly, which leads to a sharp increase in the shear plastic viscosity of concrete. Han, et al. [44] showed that the pumpability of concrete increased with the increase of air content. It is inferred that the increase of liquid surface tension under LAP can reduce air content, which increases the viscosity of concrete, and makes it difficult for the concrete to flow. All these can decrease the pumpability of cement concrete mixture.

#### 3.1.4. Workability of Concrete Mixture

Liu [2] studied the relationship between slump and air content in cement concrete. The results show that increasing the air content of cement concrete can improve its workability. The research results of Sun [45], Zhang, et al. [46] and Zhou, et al. [47] are also consistent with those of Liu [2]. There are many uniformly-distributed and independent spherical micro-bubbles in concrete under normal AP. This improves the workability of concrete mixtures (the visual property is the change of slump or VB consistency). The increase of air content makes the uniformity and cohesion of concrete increase. Bubble bonding with solid particles can reduce the tendency of its subsidence, while it also reduces the flow of water, which improves the bleeding and segregation of concrete. Therefore, workability of concrete can decrease due to the decrease of air content under LAP.

To sum up, air content is the key factor that affects the workability of concrete. Under an LAP condition, the surface tension of a liquid increases, which results in a difficulty in bubble introduction and bubble stability. The air content of concrete is seriously affected. As a result, the workability of the concrete can decrease. The main manifestations are that the slump and the slump flow of cement concrete are affected, the pumpability of cement concrete decreases, and the workability of the cement concrete mixture decreases.

### 3.2. Influence of LAP on Mechanical Properties of Concrete

#### 3.2.1. Compressive Strength of Concrete

The air content in concrete is not the only parameter affecting compressive strength [32,48,49,50,51,52,53], since pore structure can also affect the strength of concrete. Therefore, the change of strength can be analyzed through the distribution of air content and pore structure. At present, the pore structure characteristics of concrete are characterized by three parameters: The bubble spacing coefficient; bubble specific surface area, and bubble average radius.

Ma [11] showed that the compressive strength of concrete decreased with the decrease of AP when the humidity was constant during the curing process. Under LAP, the boiling point of water decreases, which accelerates the evaporation of moisture. In the process of setting and hardening of fresh concrete, moisture evaporation and humidity field accelerate the outward migration of internal moisture. This causes the bubble to escape and crack with the migration of moisture, which reduces the air content, and increases the average radius and spacing coefficient of bubbles. Therefore, the pore structure changes, and its macroscopic property is reflected in the reduction of compressive strength.

Liu [2] showed that the reduction of compressive strength of concrete under a normal AP was not only related to air content, but was also related to pore structure. When the average pore size was small, the reduction rate of compressive strength was also small. Zhou, et al. [47] also made similar findings. They discovered that when the air content of concrete was less than 5% under normal AP, the correlation between compressive strength and air content varied greatly at 3d and 7d curing ages.

According to the results of the above two researchers, the compressive strength and air content of concrete are related to pore structures. The fresh concrete is unstable in thermodynamics under LAP. The air content decreased significantly. The pore structure characteristics of hardened concrete change (bubble spacing coefficient increases; the number of bubbles per unit volume is small; the specific surface area and average bubble radius both increase), which affects the compressive strength [1,5,6,7,32]. Specifically, the content of bubbles in concrete is too small to play a buffer role in the structure under AP action, so that the compressive strength decreases. In addition, LAP has an effect on cement hydration. The early strength of concrete at 3d and 7d curing ages is mainly affected by the hydration degree of cementitious materials. In addition, LAP accelerates the outward movement of moisture, which leads to an incomplete hydration of the concrete [54,55]. It increases porosity and average pore size, and affects the density of concrete, which can decrease the compressive strength of cement concrete.

#### 3.2.2. Flexural Strength of Concrete

Ma [11] showed that the flexural strength of concrete decreased with the decrease of AP when the humidity was constant during the curing process. The LAP accelerates the evaporation of moisture on the surface of concrete, which causes microcracks in hardened concrete. The reduction of hydration products leads to the decrease of caking property between aggregate and cement paste, which makes it difficult for aggregate to wrap bubbles. Bubbles crack easily because they are not protected by the wrapped aggregate. Therefore, there are not enough bubbles to uniformly distribute inside the concrete to act as a buffer and relieve the appearance of cracks, which results in the decrease of the flexural strength of cement concrete.

Liu [2] showed that bubbles could reduce the internal microcracks formed by concrete during hardening. Compressive strength was less sensitive to these microcracks, but flexural strength was very sensitive. It can be concluded that the structure of cement concrete is easy to be loose, and the bubble characteristics change unfavorably under LAP. Harmful porosity of cement concrete increases, and microcracks are formed under freezing and thawing cycles. Therefore, LAP greatly affects the flexural strength of cement concrete.

According to the results of the above researchers, this work is summarized in Table 9. To sum up, the mechanical properties of cement concrete is closely related to bubbles. Among them, the bubble radius has a good correlation with the compressive strength. The bubble characteristics under LAP are worse than those under normal AP. Therefore, the mechanical properties of cement concrete will be significantly affected by LAP. The main manifestations are that the compressive strength of cement concrete decreases, and the flexural strength of cement concrete also decreases.

### 3.3. Effect of LAP on Durability of Concrete

#### 3.3.1. Frost Resistance of Concrete

Some researchers [56,57,58,59] proved that bubbles were related to frost resistance of concrete. In addition, some studies showed that the spacing coefficient and specific surface area of the bubble were usually used to determine the frost resistance of concrete [60]. Concrete with a smaller spacing coefficient and higher specific surface area had better frost resistance. The bubble spacing is the main factor [61,62,63]. The frost resistance of concrete is expressed by a durability coefficient (Equation (7)) and the durability coefficient decreases obviously with the increase of bubble spacing.

(7) $$K_{n}=PN/300$$

where, the *K_n_* is durability coefficient; the *N* is the frequency of freeze-thaw cycle; the *P* is relative dynamic elastic modulus after N freeze-thaw cycles.

Li, et al. [7] showed that the air content of concrete decreased about 20%–49% in contrast to that in normal AP when AP decreased to 50 kPa. It directly affected the frost resistance of the concrete. The existence of free pore water in cement paste is the main cause of any freezing damage. The porosity of concrete increases under LAP, which makes the free water increase. The free water in pores constantly produces pressure on the pore wall after repeated freezing and thawing. Yan, et al. [32] showed that the concrete specimens in the Hubei area had a higher relative elastic modulus and smaller mass loss after 150 freeze-thaw cycles. Its frost resistance was better than that of the Tibet area after 150 freeze-thaw cycles. The results are shown in Table 10 [32].

As shown in Table 10 [32], the AP in the Tibet area is lower than that in the Hubei area, China. Therefore, the air content of concrete in Tibet is lower than that in Hubei. After 150 freeze-thaw cycles, the bubble characteristics in concrete in the Tibet area gradually deteriorated. The relative elastic modulus of specimens in the area of Tibet is lower than that of those in the Hubei area. The mass loss of Tibet area specimens is higher than those of the Hubei area. Therefore, the frost resistance of concrete under LAP is much lower than that under normal AP.

Wang, et al. [64] showed that air content could improve the frost resistance of concrete within a reasonable range (3.8%–5.6%). Liu [2], Gong, et al. [65], Liu, et al. [66] and Dai, et al. [67] have similar conclusions. They show that the frost resistance and durability of concrete can be improved by properly increasing the air content of the same concrete. It can be seen that the air content has an effect upon the frost resistance of concrete. However, the air content in concrete can obviously decrease, and the porosity of concrete can increase under LAP. This can cause more pore water to freeze and expand to form an expansion pressure. Because there is not enough bubble acting as the expansion space to relieve the pressure of the cement paste, the bubble cannot play any role in alleviating the pressure, which makes the frost resistance of the concrete decrease.

#### 3.3.2. Impermeability of Concrete

Giergiczny, et al. [68], Han, et al. [69], Zhang, et al. [70] and Zoubeir, et al. [71] showed that bubbles had an effect on the impermeability of concrete. According to the effect mentioned above of LAP on bubbles, the study of bubbles under LAP is the key to exploring the effect of LAP on impermeability of concrete. Ma [11] showed that the impermeability of concrete decreased with the decrease of AP, when the humidity was constant during the curing process. LAP accelerates the migration of moisture, which makes bubbles escape with this moisture migration. The air content decreases; the porosity increases; the average pore size increases; and the volume of gel pores decreases. Therefore, the connectivity of pores increases, which makes Cl^−^ invade concrete easily [72,73] and decreases the impermeability of concrete.

Liu [2] showed that the appropriate air content could improve the impermeability of concrete under normal AP. Gong, et al. [65], Dai, et al. [67] and other researches [74,75,76] also have a consistent conclusion. However, LAP significantly reduces air content, which makes bubbles not able to resist osmotic pressure. Under the impact of AP, concrete cracks easily, and concrete structures deteriorate [77]. In addition, bubble distribution is not uniform, and the reduction of repulsion between bubbles under LAP, which makes the thickness of bubble film become thin and easy to crack. Therefore, the pore passages of concrete increase. Water and other substances are easy to infiltrate into concrete, which makes its impermeability decrease.

According to the studies of the above researchers, this work is summarized in Table 11. To sum up, bubble characteristics are the key to be able to determine the effect of LAP on the durability of cement concrete. Among them, the bubble spacing coefficient has a good correlation with frost resistance. Under LAP, the stability of bubbles and the characteristics of bubbles are deteriorated, which affects the durability of cement concrete. The main manifestations are the frost resistance and the impermeability of cement concrete decreases.

## 4. Improvement Techniques

### 4.1. Mechanism for Developing New AEA

**Figure 7:**
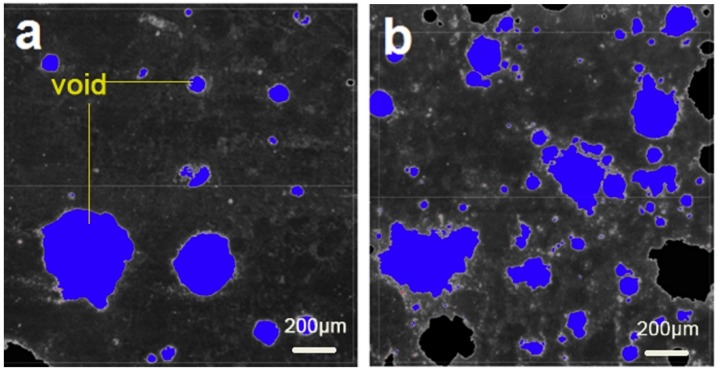
Void images of hardened cement mortar: (**a**) no AEA, (**b**) with AEA.

According to the results [78,79], bubbles in cement mortar without AEA are rare and unstable, and much smaller bubbles can be produced in cement mortar with AEA, as shown in Figure 7 [78]. As we know, entraining a large number of uniform and tiny voids is more beneficial for both the workability and durability of cement concrete [80,81,82,83]. However, LAP seriously weakens the properties of AEA. Therefore, it is necessary to develop a new AEA. The AEA is mainly a mixture of various surfactants. Its foaming capacity and foam stability mainly depend upon the adsorption and assembly of the hydrophilic and hydrophobic chains of surfactant molecules at the water-air interface [80,84,85]. However, the LAP environment in plateau areas greatly limits its surface activity. Therefore, this work puts forward the idea and mechanism for developing new AEA. We can add particles with opposite charges to the ionic oligomeric surfactants as a new AEA, such as nano-silica (with negative charges), coexisting with cationic oligomeric surfactants, producing synergistic effects in both foaming capacity and foam stability.

**Figure 8:**
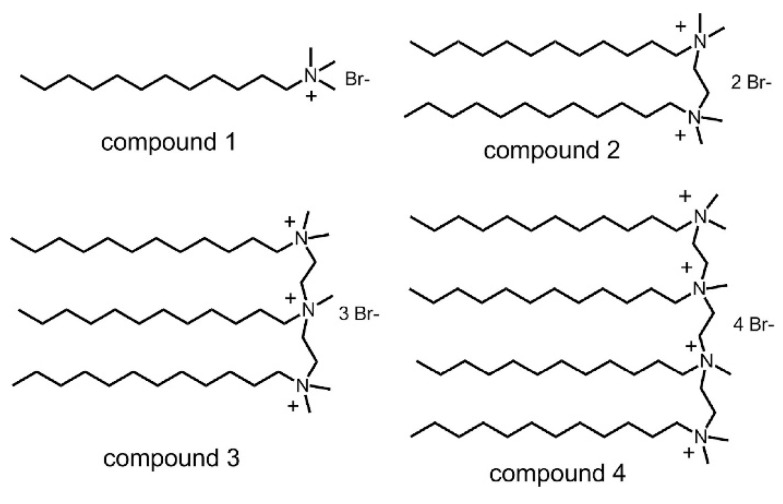
Chemical structures of the cationic oligomeric surfactants (compounds 2–4) and the corresponding monomeric analog (compound 1).

In such systems, in-situ hydrophobicity occurs, that is, surfactant molecules with opposite charges are adsorbed to the surface of particles by electrostatic attraction, forming a monolayer, making the surface of particles hydrophobic, so that particles can be adsorbed to the water-air interface to stabilize bubbles. Oligomeric surfactants can be regarded as oligomers of traditional monomeric surfactant fragments, which are linked by spacer groups near their ionic groups [86,87,88,89,90,91,92]. Figure 8 [79] shows the chemical structures of the cationic oligomeric surfactants [79]. Since ionic groups of surfactant molecules are covalently coupled, the electrostatic repulsion among ionic groups is effectively neutralized, and the distance among hydrophobic chains is greatly shortened. As some results [93,94,95,96], the assembly of oligomeric surfactants on the water-air interface is more compact and orderly, and some unique properties including lower critical micelle concentration (CMC), higher surface activity and higher foaming capacity, and its effect on strength, is also low. For example, adding nano-silica to cationic oligomeric surfactant can not only produces synergistic effects in both foaming capacity and foam stability, but also promotes cement secondary hydration to increase concrete strength.

### 4.2. Proper Adjustment of Preparation Process

Method, time and temperature for concrete preparation should be mentioned. According to the results in references [30,31], if the ordinary method is used to mix the concrete, the cumulative volume ratio of the pore diameter greater than 10 μm is the highest, which seriously affects the mechanical properties and durability of the concrete. If the vibration stirring is used to mix the concrete, the pore size distribution and pore distribution can be significantly improved. The pore diameter of cement concrete can decrease. The macropore numbers decrease, and the small pore numbers increase. This indicates that vibration stirring only increases capillary porosity and gel porosity, while also increasing air content without increasing macropores. Therefore, the strength and durability of cement concrete are improved. In addition, adding AEA can improve the pore gradation of cement concrete. The ratio of small pores increases, and the ratio of macropores decreases. Most of the pore diameters are between 1 μm and 10 μm, which fully shows that AEA can effectively improve the durability of cement concrete. However, pores close to 10 μm are significantly increased, which leads to the loss of strength. Therefore, cement concrete is prepared by combining vibration stirring with AEA.

The cement concrete prepared under LAP is in an unstable state of thermodynamics. The bubble film thickness is thin, and the bubble stability is poor. Excessive vibration stirring or excessive vibration time can result in more loss of bubbles. Therefore, under the precondition of satisfying compactness, the shortest time of vibration stirring is used to ensure that the cement concrete has enough air content to improve the properties of concrete. The temperature difference under LAP in plateau areas is high. The high temperature increases the surface tension of the liquid film of bubbles, and decreases the thickness and strength of the liquid film, which makes bubbles crack easily. Therefore, the temperature and bubble characteristics should be controlled, in order to ensure the uniform distribution and stability of bubbles.

### 4.3. Adoption of Reasonable Maintenance Methods

Under LAP conditions, bubbles in fresh cement concrete have been in an unstable state. The bubble characteristics in hardened cement concrete are changed. In addition, LAP accelerates the evaporation of internal and surface moisture in cement concrete. During the process of moisture migration, the bubble spacing coefficient increases; the bubbles’ number per unit volume decreases; the bubble distribution is uneven; and the pore structure deteriorates. Therefore, attention should be paid to the maintenance of cement concrete. Moisture should be supplied to concrete in time to prevent pores changing due to the evaporation of moisture. The internal and external maintenance methods can be used. They play a dual role of internal water retention and external water resistance of cement concrete, which effectively ensures the full hydration of cement materials. It promotes property improvements of cement concrete.

To sum up, when cement concrete is prepared under LAP, the changes of bubble characteristics should always be paid attention to. The adoption and the development of AEA, the mixing methods, time and temperature and maintenance methods should also be paid great attention. These methods can be adopted to improve the bubble characteristics to ensure the properties of cement concrete.

## 5. Conclusions and Suggestions

In summary, the LAP environment in plateau areas is a kind of environmental condition which is easy to be neglected. Its influence on the properties of cement concrete is mainly reflected by the bubble characteristics. Therefore, this work mainly analyzes and summarizes the property changes of cement concrete through the influence of LAP upon bubble characteristics. In view of these influences, corresponding improvement techniques are put forward. Some opinions on the mechanism for developing new AEA were put forward, which were beneficial to the development of AEA. The following conclusions can be drawn:

(1) The air content in cement concrete is mainly introduced by AEA. In plateau areas, LAP increases the surface tension of the liquid, while AEA introduce bubbles by reducing the surface tension of the liquid. It leads to the fact that the AEA under LAP is not only difficult to foam, but also poor in foam stability. Therefore, LAP significantly weakens the effects of AEA, which reduces air content in cement concrete.

(2) The bubble is the key factor affecting the workability of the cement concrete mixture. Under LAP, the decrease of air bubbles may increase the friction or decrease the adhesion between aggregates, which makes it difficult to mix well, and results in the decrease of the workability of cement concrete.

(3) The mechanical properties of cement concrete are closely related to the bubbles therein. The bubble characteristics are deteriorated under LAP. Bubbles are not enough to buffer pressure, which affects the internal structure of cement concrete. In addition, LAP accelerates the moisture movement in cement concrete. On the one hand, it causes incomplete hydration and increases harmful pores. On the other hand, the process of moisture migration leads to the increase of pores in cement concrete, which affects the mechanical properties of cement concrete.

(4) Bubbles affect the durability of cement concrete. The changes of bubble characteristics should be mainly considered. Under LAP, the bubble film becomes thin; the strength of the bubble decreases; the proportion of large diameter bubbles increases. As a result, the spacing coefficient and the specific surface area of the bubble increases, which can decrease the durability of concrete. It is suggested that the spacing coefficient of the bubble be used instead of air content to improve frost resistance of concrete.

(5) The development of new AEA should be concerned. This work recommends the addition of particles with opposite charges to the ionic oligomeric surfactants as a new AEA, such as nano-silica (with negative charges), coexisting with cationic oligomeric surfactants.

(6) Finally, it is hoped that further research on the correlation between the bubble characteristics and cement concrete properties can be carried out in order to accurately reflect the changes of cement concrete properties under LAP. At the same time, it is urgently hoped that new types of AEA for LAP can be developed as soon as possible.

## Figures and Tables

**Table 1:** Foaming results in Beijing and Golmud of China.

Areas	Air Entraining Agent Quantity (g)	Maximum Bubble Volume (mL)	Foam Duration (h)	Bubble Shape
Beijing	0.2	11	27	Small particle size, large quantity
Golmud	0.2	10	15.2	Large particle size, small quantity

Note: 1. The solution concentration is the air entraining agent, in which the above weight is dissolved in 100 mL of water, respectively. 2. The duration of the foam is the time from the end of the shaking to the disappearance of the foam to expose the horizontal liquid level.

**Table 2:** Foaming ability of air entraining agent (AEA) under different atmospheric pressures (AP).

Sample	AP (kPa)	Surface Tension		Foaming Ability				
		Test Value (mN·m^−1^)	Growth Rate (%)	Foaming Capacity (mL)	Foaming Capacity at 3 min (mL)	Foam Stability (%)	Defoaming Time (h)	Bubble Shape
AEA-A	101.1	29.4	100	Full	Full	100	≥72 h	Small
	65.9	32.9	112	Full	Full	100	≥48 h	Much big
	57.2	34.6	118	50	49	98	≥48 h	Little, sparse
AEA-B	101.1	31.9	100	38	36	95	≥36 h	Moderate
	65.9	34.2	107	32	30	94	≥24 h	Much big
	57.2	36.3	114	29	26	90	≥24 h	Little, sparse

Note: AEA-A, synthetic anionic surfactant air entraining agent; AEA-B, alkyl sulfonate air entraining agent; Full, bubbles fill measuring cylinder.

**Table 3:** Relative air content of concrete after normalized treatment.

AEA	Initial Air Content (%)	AP (kPa)								
		80			60			50		
		1^#^	2^#^	3^#^	1^#^	2^#^	3^#^	1^#^	2^#^	3^#^
Saponins	3	0.94	0.94	0.88	0.84	0.82	0.81	0.81	0.76	0.75
	5	0.87	0.81	0.83	0.75	0.72	0.69	0.66	0.61	0.62
	7	0.86	0.81	0.81	0.72	0.64	0.64	0.61	0.53	0.53
JDU	3	0.88	0.88	0.87	0.76	0.78	0.80	0.70	0.69	0.73
	5	0.87	0.82	0.77	0.73	0.70	0.62	0.63	0.62	0.55
	7	0.83	0.82	0.76	0.71	0.65	0.60	0.60	0.56	0.51
Rosin	3	0.91	0.85	0.90	0.84	0.76	0.83	0.75	0.65	0.73
	5	0.82	0.77	0.80	0.69	0.63	0.64	0.65	0.62	0.62
	7	0.74	0.79	0.75	0.66	0.64	0.59	0.62	0.61	0.55
Polyether	3	0.85	0.90	0.90	0.82	0.81	0.87	0.82	0.74	0.80
	5	0.82	0.75	0.78	0.76	0.67	0.67	0.74	0.67	0.61
	7	0.74	0.77	0.71	0.69	0.70	0.64	0.67	0.68	0.61

**Table 4:** The decrease of air content under low atmospheric pressure (LAP) (%).

Initial Air Content (%)	Air Entraining Agent Type			
	Saponins	JDU	Rosin	Polyether
3	6-5	12-31	9-35	10-20
5	13-39	13-45	18-38	18-39
7	14-47	17-49	21-45	23-39

Note: The reduced value in the table corresponds to the low atmospheric pressure (LAP) range of 50–80 kPa. The lower the AP, the more the reduced value.

**Table 5:** Influence of low atmospheric pressure (LAP) on AEA effect in concrete.

Researchers	Test	Results	Summary
Zhu, et al. [3]	Foaming test Property test	The AP affects the air entraining ability of AEA, which ultimately leads to a decrease in the air content of concrete under LAP.	From different researchers and tests, we know that the LAP significantly reduces the foaming capacity and foam stability of AEA. It can affect the air content of concrete, which is not conducive to the properties of concrete.
Yan, et al. [32]	AEA quality test		
Li, et al. [4,5,8,33]	Simulation test of LAP Porosity test	LAP can significantly reduce the air entraining ability of AEA. No matter what kind of AEA is added, the air content of concrete decreases with the decrease of AP. When other conditions are constant, the air content of concrete decreases linearly with the decrease of AP. When initial air content of concretes is high, the reduction rate of air content increases with the decrease of AP. When the initial air content of concretes is similar, the greater the slump of concrete, the stronger its resistance to the decrease of air content caused by the decrease of AP.	
Ke, et al. [9,10]	Pumpability test		

**Table 6:** Bubble characteristics of hardened concrete under different AP (equivalent dosage of air entraining agent).

AEA	Rosin		JDU		Saponins		Polyether	
	LAP	AP	LAP	AP	LAP	AP	LAP	AP
Air content of hardened concrete (%)	2.5	4.6	2.54	4.87	3.03	4.92	2.95	4.92
Bubble spacing coefficient (μm)	337	207	358	175	313	184	316	178
Specific surface area of stomata (mm^−1^)	20.44	25.87	19.47	29.79	20.62	31.21	20.64	29.01
Average bubble diameter (μm)	196	155	205	134	194	128	194	138
Number of bubbles per unit volume	308	718	298	875	377	927	367	862

**Table 7:** Bubble characteristics of hardened concrete under different AP (similar air content).

AEA	Rosin		JDU		Saponins		Polyether	
	LAP	AP	LAP	AP	LAP	AP	LAP	AP
Air content of hardened concrete (%)	3.86	3.62	4.47	4.46	3.87	3.9	3.81	3.72
Bubble spacing coefficient (μm)	266	177	212	119	203	157	319	178
Specific surface area of stomata (mm^−1^)	21.8	25.96	25.59	39.66	28.25	36.55	18.26	32.95
Average bubble diameter (μm)	184	171	156	138	142	109	219	121
Number of bubbles per unit volume	508	823	691	929	660	861	420	740

**Table 8:** Effect of different humidity (H) on the pore structure of concrete under different pressure.

Curing Ages	Curing Condition		Porosity (%)		Total Mercury Intake (mL/g)		Average Pore Size (nm)	
3d	P_50_H_30_ P_75_H_30_	P_50_H_60_ P_75_H_60_	14.86 14.25	13.87 13.12	0.0838 0.0833	0.0748 0.0729	53.3 46.7	38.5 35.4
7d	P_50_H_30_ P_75_H_30_	P_50_H_60_ P_75_H_60_	14.07 13.08	13.03 12.25	0.0805 0.079	0.0733 0.0711	38.7 35.2	34.1 31.6
28d	P_100_H_98_		12.79	12.79	0.0689	0.0689	20.5	20.5

Note: The subscript of the P is the AP value; The subscript of the H is the humidity.

**Table 9:** Influence of normal AP / LAP on the mechanical properties of concrete.

Researchers	Mechanical Properties	Results	Summary
Ma [11]	Compressive strength	During the curing process, when humidity is constant, the compressive strength of concrete decreases with the decrease of AP.	From different researchers and tests, we know that the LAP affects the mechanical properties of concrete by the bubble characteristics (especially air content).
	Flexural strength	During the curing process, when the humidity is constant, the flexural strength of concrete decreases with the decrease of AP.	
Liu [2]	Compressive strength	Under normal AP, the compressive strength of concrete is related to the air content and pore structure. When the average pore size is small, the reduction rate of compressive strength is also small. On the contrary, the average pore size increases under LAP, which increases the reduction rate of compressive strength.	
	Flexural strength	Under AP, bubbles can reduce the internal microcracks formed by concrete during hardening. Flexural strength is more sensitive to these micro cracks than compressive strength. On the contrary, under LAP, the air content decreases. There are not enough bubbles to inhibit microcracks. Therefore, LAP greatly affects the flexural strength.	
Zhou, et al. [47]	Compressive strength	Under AP, when air content of concrete was less than 5%, the correlation between compressive strength and air content varied greatly at 3d and 7d curing ages. Therefore, under LAP, the air content decreases, which greatly affects compressive strength of concrete.	

**Table 10:** Frost resistance of concrete formed under different AP.

AP (kPa)	AEA Dosage (%)	Air Content (%)	Frost Resistance at 28-Day Curing Ages					
			Relative Dynamic Elasticity Modulus (%)			Loss of Mass (%)		
			P_50_	P_100_	P_1__50_	W_50_	W_100_	W_150_
Tibet, 65.9	0.04	3.6	90	86	78	0.4	1.2	2.7
Hubei, 101.1	0.04	5.1	93	90	84	0	0.3	1.0

**Table 11:** Influence of normal AP / LAP on the durability of concrete.

Researchers	Durability	Results	Summary
Li, et al. [7]	Frost resistance	When the AP decreases to 50 kPa, the air content of concrete decreases about 20%–49% in comparison to that in normal AP. It directly affects the frost resistance of the concrete.	From different researchers and tests, we know that the LAP affects the durability of concrete by the bubble characteristics (especially air content).
Yan, et al. [32]	Frost resistance	After 150 freeze-thaw cycles, the concrete specimens in the Hubei area have higher relative elastic modulus and smaller mass loss. Its frost resistance is better than that of Tibet.	
Liu [2], Wang, et al. [64], Gong, et al. [65], Liu, et al. [66], Dai, et al. [67]	Frost resistance	Under normal AP, proper air content can improve the frost resistance of concrete. However, LAP can significantly reduce air content, which greatly affects the frost resistance.	
Ma [11]	Impermeability	The impermeability of concrete decreases with the decrease of AP when the humidity is constant during the curing ages.	
Liu [2], Gong, et al. [65], Dai, et al. [67]	Impermeability	Under normal AP, proper air content can improve the impermeability of concrete. However, LAP can significantly reduce air content, which greatly affects the impermeability.	
